# A Personalized Avatar-Based Web Application to Help People Understand How Social Distancing Can Reduce the Spread of COVID-19: Cross-sectional, Observational, Pre-Post Study

**DOI:** 10.2196/38430

**Published:** 2023-04-25

**Authors:** Doriane Etienne, Patrick Archambault, Donovan Aziaka, Selma Chipenda-Dansokho, Eve Dubé, Catherine S Fallon, Hina Hakim, Jason Kindrachuk, Dan Krecoum, Shannon E MacDonald, Ruth Ndjaboue, Magniol Noubi, Jean-Sébastien Paquette, Elizabeth Parent, Holly O Witteman

**Affiliations:** 1 VITAM – Centre de recherche en santé durable Université Laval Québec, QC Canada; 2 Department of Emergency Medicine Centre intégré de santé et de services sociaux de Chaudière-Appalaches Québec, QC Canada; 3 Department of Family Medicine and Emergency Medicine Faculty of Medicine Université Laval Québec, QC Canada; 4 Department of Anthropology Université Laval Québec, QC Canada; 5 Laboratory of Emerging Viruses Department of Medical Microbiology & Infectious Diseases University of Manitoba Winnipeg, MB Canada; 6 Faculty of Nursing & School of Public Health University of Alberta Edmonton, AB Canada; 7 Research Center on Aging Centre intégré universitaire de santé et de services sociaux de l'Estrie - CHU de Sherbrooke Université de Sherbrooke Sherbrooke, QC Canada; 8 Research Centre of the CHU de Québec Laval University Québec, QC Canada

**Keywords:** social distancing, COVID-19, SARS-CoV-2, pandemic, personalized risk communication, public health, digital health intervention, web application, visualization, personalized avatar

## Abstract

**Background:**

To reduce the transmission of SARS-CoV-2 and the associated spread of COVID-19, many jurisdictions around the world imposed mandatory or recommended social or physical distancing. As a result, at the beginning of the pandemic, various communication materials appeared online to promote distancing. Explanations of the science underlying these mandates or recommendations were either highly technical or highly simplified.

**Objective:**

This study aimed to understand the effects of a dynamic visualization on distancing. Our overall aim was to help people understand the dynamics of the spread of COVID-19 in their community and the implications of their own behavior for themselves, those around them, the health care system, and society.

**Methods:**

Using Scrum, which is an agile framework; JavaScript (Vue.js framework); and code already developed for risk communication in another context of infectious disease transmission, we rapidly developed a new personalized web application. In our application, people make avatars that represent themselves and the people around them. These avatars are integrated into a 3-minute animation illustrating an epidemiological model for COVID-19 transmission, showing the differences in transmission with and without distancing. During the animation, the narration explains the science of how distancing reduces the transmission of COVID-19 in plain language in English or French. The application offers full captions to complement the narration and a descriptive transcript for people using screen readers. We used Google Analytics to collect standard usage statistics. A brief, anonymous, optional survey also collected self-reported distancing behaviors and intentions in the previous and coming weeks, respectively. We launched and disseminated the application on Twitter and Facebook on April 8, 2020, and April 9, 2020.

**Results:**

After 26 days, the application received 3588 unique hits from 82 countries. The optional survey at the end of the application collected 182 responses. Among this small subsample of users, survey respondents were nearly (170/177, 96%) already practicing distancing and indicated that they intended to practice distancing in the coming week (172/177, 97.2%). Among the small minority of people (n=7) who indicated that they had not been previously practicing distancing, 2 (29%) reported that they would practice distancing in the week to come.

**Conclusions:**

We developed a web application to help people understand the relationship between individual-level behavior and population-level effects in the context of an infectious disease spread. This study also demonstrates how agile development can be used to quickly create personalized risk messages for public health issues like a pandemic. The nonrandomized design of this rapid study prevents us from concluding the application’s effectiveness; however, results thus far suggest that avatar-based visualizations may help people understand their role in infectious disease transmission.

## Introduction

Around the world, COVID-19 responses included messaging and interventions in digital media spaces [1,2]. To reduce the transmission of SARS-CoV-2 and the associated spread of COVID-19, many jurisdictions around the world imposed mandatory or recommended social or physical distancing (hereafter, “distancing”) [3]. Distancing is a measure that helps reduce the spread of highly contagious respiratory viruses when a potential transmission-reducing vaccine is not available. Distancing not only limits contact or the frequency of gathering in public places [4-6] but also reduces the increase in the number of cases in a community, thereby reducing or controlling the potential burden on health systems [7]. As a result, at the beginning of the pandemic, various communication materials appeared online to promote distancing [8].

Implementing distancing is only possible with public cooperation [9,10]. Such cooperation requires that people understand what they are being asked to do and why so that they adopt appropriate behaviors to help manage the pandemic [11,12]. Effective public communication in times of crisis should be balanced to reduce negative factors such as anxiety or misinformation while promoting cooperation with recommendations [13,14]. Public health messages must also be tailored to address public concerns and speak to all members of the public across diverse contexts, backgrounds, and levels of understanding [15]. A rapid systematic review of the determinants of preventive behavior for viruses similar to COVID-19 found small positive correlations between perceptions of severity, threat, efficacy, and the adoption of distancing behavior. However, for those concerned, anxious, or worried about their relatives and themselves being ill, the correlation between the adoption of distancing behavior and emotions was moderate to strong. The evidence from this review suggests that a focus on awareness of the impact of COVID-19 on the individual or their surroundings may assist in promoting distancing [12].

With these considerations in mind, at the beginning of the COVID-19 pandemic, we observed that early digital risk communication materials shared on social media explaining the science of distancing either introduced distancing in a highly technical way or in an overly simplified way [16]. There appeared to be fewer communication materials in the middle ground between these extremes that would be suitable across levels of health literacy to help promote distancing [17]. Hart and colleagues [18] described how the following 3 behavioral science levers may promote distancing: (1) demonstrate: show what distancing is by explaining what to do and making it memorable; (2) enable: make distancing easy, and offer reliable sources of information that address recommendations; (3) motivate and prompt: provide information about the consequences of distancing or of catching COVID-19.

We therefore aimed to create a digital risk communication tool using these levers. Specifically, we adapted a personalized avatar-based web application to show the impact of distancing on COVID-19 transmission in a person’s community. Our study extends previous research by Hakim and colleagues [19], who developed a web application to communicate epidemiological evidence on herd immunity through an animated visualization using personalized avatars. Designed to optimize users' cognitive and emotional responses, this application demonstrated the potential to communicate the relationship between individual behavior and community health. In this animation, the user builds a personalized avatar and 8 other avatars representing family, friends, or colleagues. The application then integrates these avatars into a 2-minute visualization showing how different parameters (eg, vaccine coverage, contacts within the community) influence herd immunity. The application aims to incorporate emotional considerations around infectious disease transmission, such as concern for people around oneself who may be more vulnerable to infection or severe outcomes [19].

Throughout the pandemic, the availability of clear and transparent information on the evolution of the scientific evidence has proved critical for building (or damaging) trust in institutions and empowering the public to better understand the situation and make evidence-based decisions. This cross-sectional, observational, pre-post study therefore aimed to understand the impact of a dynamic visualization on distancing during the early months of the COVID-19 pandemic. Our overall aim was to help people understand the dynamics of the spread of COVID-19 in their community and the implications of their own behavior for themselves, those around them, the health care system, and society. This paper describes the process of developing the web application and the associated study examining its effects on distancing and its usage metrics.

## Methods

### Storyboard and Script

Based on a previous herd immunity or community immunity web application [19], our multidisciplinary team developed a storyboard and narration script to explain distancing in both English and French (Multimedia Appendix 1).

### User Flow

**Figure 1:**
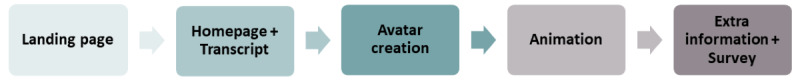
User flow representation.

To build our web application, we started by modeling the new navigation flow (Figure 1) [20]. First, a landing page informs users that the web application is optimized for the majority of browsers, except for Internet Explorer and Microsoft Edge, which are not supported. Users are directed to the home page, where they can select their preferred language (English or French) and access a descriptive transcript for people using screen readers (eg, people who are blind or vision-impaired) or with low bandwidth (eg, people who live in locations with limited internet service.) Next, users are directed to the avatar creation page. Once their avatar is created, users can launch a narrated animation featuring their personalized avatar. At the end of the animation, users receive additional information with links to Canadian government and international websites about COVID-19 prevention and are invited to complete a brief survey.

### Web Application Development

To develop our web application, we used Scrum, which is an agile framework [21-23], and JavaScript (Vue.js framework) as our programming language [24]. Agile practices may be more pragmatic than waterfall software development methods, as they promote adaptive planning, evolutionary development, rapid delivery, and continuous improvement [25-27]. In keeping with our agile approach, we performed short iterations that allowed us to quickly adapt the prototype, adding new features and fixing bugs. By building on existing code and using a rapid development approach, in 3 weeks, we created a web application that respected accessibility standards, including providing descriptive text for people who are blind or vision-impaired and captions for people who are deaf or hearing-impaired.

### The Web Application

**Figure 2:**
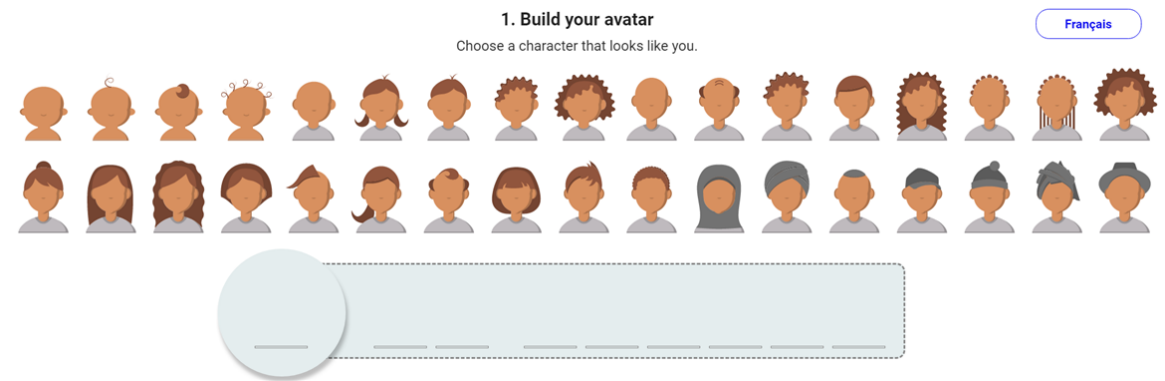
Avatar creation screen.

In our web application, people create avatars (Figure 2) that represent themselves and people around them by choosing among a set of avatars, then adjusting skin color, hair color, glasses, facial hair, and head covering accessories such as a hijab, turban, and caps, as desired.

**Figure 3:**
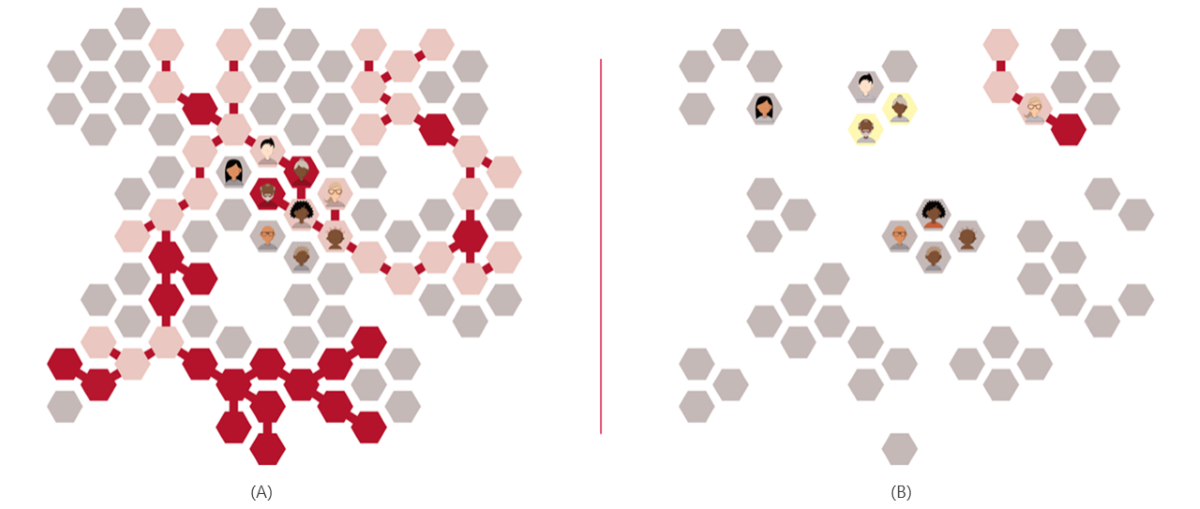
Differences in transmission (A) without distancing and (B) with distancing.

These avatars are used in a 3-minute animation illustrating an infectious disease epidemiological model of COVID-19 [28], showing the differences in transmission with and without distancing (Figure 3). During the animation, the narration explains the science of how distancing reduces the transmission of COVID-19 in plain language. The web application offers complete captions for those who may be unable to hear the narration or who prefer to read it and a full written descriptive version for people who may be unable to see the visualization, for example, people using screen readers. At the end of the animation, we offered viewers a summary page on distancing with links to the websites of the Public Health Agency of Canada (COVID-19 Prevention, Quarantine, and Isolation) [29] and the World Health Organization for more information [8].

### Evaluation Study Design and Data Collection

This study was a cross-sectional, observational, pre-post study in which we asked people to self-report their behavior in the week before viewing the animation and their behavioral intentions for the week to come and collected usage metrics.

#### Ethical Considerations

This study received ethical authorization from the Research Ethics Committee of Laval University (approval number 2020-098/27-03-2020). All study participants were notified in written form about the aim of the study and the fact that their participation was voluntary. They were assured of the anonymity of their data. By completing the 5-question survey, participants gave their implied free and informed consent electronically to the use of their answers in the study. Study participants were not compensated.

#### Survey

After viewing the 3-minute animation, participants were asked to complete a short, anonymous, optional 5-question survey. Participants had learned about the application after we disseminated the web application on April 8,2020, and April 9, 2020, through social media platforms. We used the lab's Twitter and Facebook accounts (@wittemanlab) and the personal Twitter account of the principal investigator, Holly Witteman (@hwitteman). The link was subsequently shared by the Twitter accounts of the Canadian Institutes of Health Research, Jane Philpott (former Canada Minister of Health), and other influential accounts. We used a convenience sample for the survey and expected to recruit about 200 participants. To be eligible for inclusion, participants had to be 18 years of age or older, able to read and understand English or French, and able to provide informed consent electronically by continuing the survey. We excluded responses from people who indicated that they could not practice distancing (for example, because they were essential workers.) We asked participants to self-report the following short set of sociodemographic characteristics: year of birth, place of residence, gender, and level of education. We also used these variables as independent variables in our analyses. We collected survey responses via Qualtrics from April 8, 2020, to May 3, 2020.

#### Application Usage

To study how people interact and generate usage metrics, we linked the web application to Google Analytics [30].

### Outcomes and Additional Data

#### Survey

Our primary outcome of interest was participants’ self-reported behavioral intentions to practice distancing in the week after viewing the web application compared with the previous week. We assessed each of these by asking participants to indicate their response on a 7-point, agree-disagree, Likert-type scale to the items, “In the past week (7 days), I practiced distancing,” and “In the coming week, I intend to practice distancing.” We included an option to indicate an inability to distance due to a public-facing occupation, “Not applicable (eg, I work in an essential service).”

#### Application Usage

From Google Analytics, we collected data about active users, sessions, technology use (device, operating system), language, and geographic location. According to the definition provided by Google, an active user is a user who has accessed the web application. At the first access of the web application, all data are stored and associated with each user. Specifically, a unique cookie stores a Google Analytics customer ID, which enables identification of the user on all devices they may use to reach the web application and to determine their number of connections [31]. Google Analytics collects descriptive data about the technologies used (eg, mobile devices) by the study population. The tracking code also enables the collection of browser data, such as the configured language, browser (eg, Chrome, Safari, Firefox), device (eg, iPad, desktop), and operating system (eg, Windows, macOS) used to access the web application. These data are updated with user activity. For each user, a time period called a session begins when the user accesses a page containing the tracking code. A session ends after 30 minutes of inaction. If the user comes back later, a new session begins. Data on language and geographic location are obtained using the ISO 639 language code [32] and ISO 3166 country code [33] set in the user’s browser.

### Analyses

#### Survey

We compared participants’ behavioral intentions to practice distancing in the week to come with their self-reported distancing behavior in the previous week. Given the exploratory nature of this study, we conducted both a paired *t* test and a Wilcoxon signed-rank test with continuity correction. Then, using ANOVA, we tested the interactions between sociodemographic data and differences between distancing intentions and self-reported distancing in the previous week. We also performed additional comparisons to explore which modalities differed using the least-squares mean method (lsmeans) [34]. We performed all statistical analyses in R, Version 3.6.2 [35]

#### Application Usage

We descriptively analyzed user data generated by Google Analytics to understand how users interacted with the web application to identify potential future improvements [30].

## Results

### Survey

#### Population

The optional survey at the end of the web application yielded 182 responses, representing 5.1% of all 3588 users. Overall, most respondents completed the optional survey in English (171/182, 94%). The average age of respondents was 42.7 (SD 15.0) years. Most respondents (111/182, 60.9%) identified as women, indicated they were university graduates (156/182, 85.7%), and reported living outside Canada (102/182, 56%; Table 1). Of the participants, 5 indicated they could not practice distancing; we conducted analyses on the remaining 177 respondents.

**Table 1:** Characteristics of respondents to the optional survey (n=182).

Responses		Results
**User language, n (%)**		
	English	171 (94)
	French	11 (6)
Age (years), mean (SD)^a^		42.7 (15)
**Place of residence, n (%)**		
	Canada — Alberta (AB)	10 (5.5)
	Canada — British Columbia (BC)	5 (2.8)
	Canada — Manitoba (MB)	0 (0)
	Canada — New Brunswick (NB)	1 (0.5)
	Canada — Newfoundland and Labrador (NL)	0 (0)
	Canada — Northwest Territories (NT)	0 (0)
	Canada — Nova Scotia (NS)	5 (2.8)
	Canada — Nunavut (NU)	0 (0)
	Canada — Ontario (ON)	44 (24.2)
	Canada — Prince Edward Island (PE)	0 (0)
	Canada — Quebec (QC)	11 (6.1)
	Canada — Saskatchewan (SK)	1 (0.5)
	Canada — Yukon (YT)	0 (0)
	Outside Canada	102 (56)
	Prefer not to answer	3 (1.6)
**Gender identity, n (%)**		
	Female	111 (61)
	Male	68 (37.5)
	Indigenous or other cultural gender minority identity (eg, 2-spirit)	1 (0.5)
	Another gender (eg, non-binary, gender-fluid)	1 (0.5)
	Prefer not to answer	1 (0.5)
**Education level, n (%)**		
	Some elementary school (completed or not)	0 (0)
	Some high school (not completed)	7 (3.8)
	High school diploma	9 (5.0)
	Apprenticeship or trade certificate or diploma	3 (1.7)
	College or polytechnical school certificate or diploma	6 (3.3)
	University degree, bachelor level or below	66 (36.3)
	University graduate degree (Master’s level)	52 (28.5)
	University graduate degree (Doctorate level)	38 (20.9)
	Do not know	0 (0)
	Prefer not to answer	1 (0.5)
**Distancing, n (%)^b^**		
	Practiced distancing in the past week	170 (96)
	Did not practice distancing in the past week	7 (4)
	Intend to practice distancing in the week to come	172 (97.2)
	No intention to practice distancing in the week to come	5 (2.8)

^a^n=174.

^b^n=177.

#### Behavioral Intentions Regarding Distancing

Among the 177 respondents, 170 (96.0%) were already practicing distancing, and 172 (97.2%) intended to practice distancing in the coming week. Of those (n=7) not yet practicing distancing in the previous week, 2 (29%) indicated, after using the web application, that they would practice distancing in the week to come. None of the people already practicing distancing indicated that they intended to change that behavior.

We found no significant difference between the respondents’ initial distancing practice (mean 7.50, SD 1.13) and intention to practice distancing in the coming week (mean 7.59, SD 1.05; t_176_= 1.87, *P*=.06; Table 2). The Wilcoxon signed-rank test (*P*=.08) was also not statistically significant.

We found a statistically significant main effect of the place of residence (*F*_7,146_=2.94, *P*=.006) and gender (*F*_1,146_=3.86, *P*=.05) on the reported practice of distancing.

**Figure 4:**
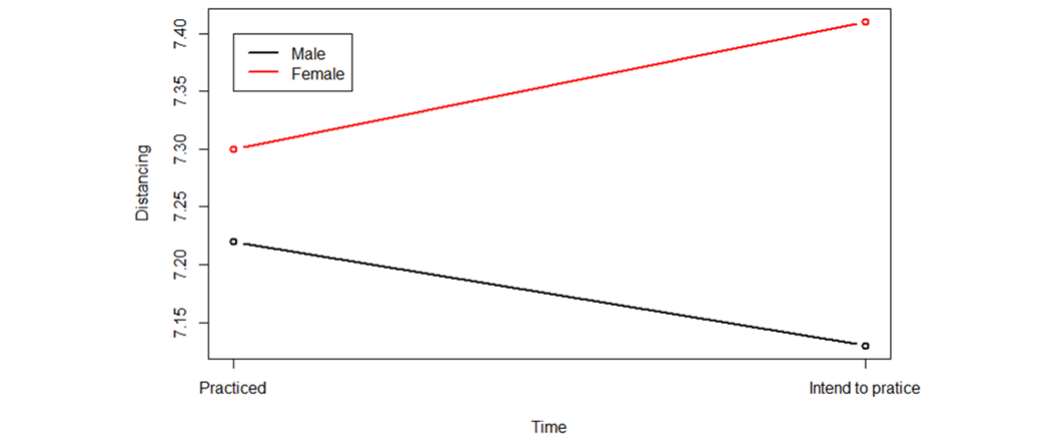
The least-squares mean related to gender interaction.

We observed a statistically significant interaction between the place of residence “outside Canada” and the difference in reported practice (t_146_=2.653, *P*=.008), indicating the application had a stronger effect on intentions to practice distancing among people living outside Canada. We also observed a statistically significant interaction by self-reported gender. Specifically, the effect of the application was positive for female respondents (t_146_=0.61, *P*=.53) and negative for male respondents (t_146_=–0.49, *P*=.62; Figure 4).

**Table 2:** Comparison of means between the initial and future practice of distancing reported after being exposed to the 3-minute animation, using a paired t test.

Response	Results, mean (SD)	*t* (df)	*P* value
Practiced distancing	7.50 (1.13)	1.87 (176)	.06
Intend to practice distancing	7.59 (1.05)		

### Application Usage

#### Users’ Characteristics and Use Pattern Data

**Figure 5:**
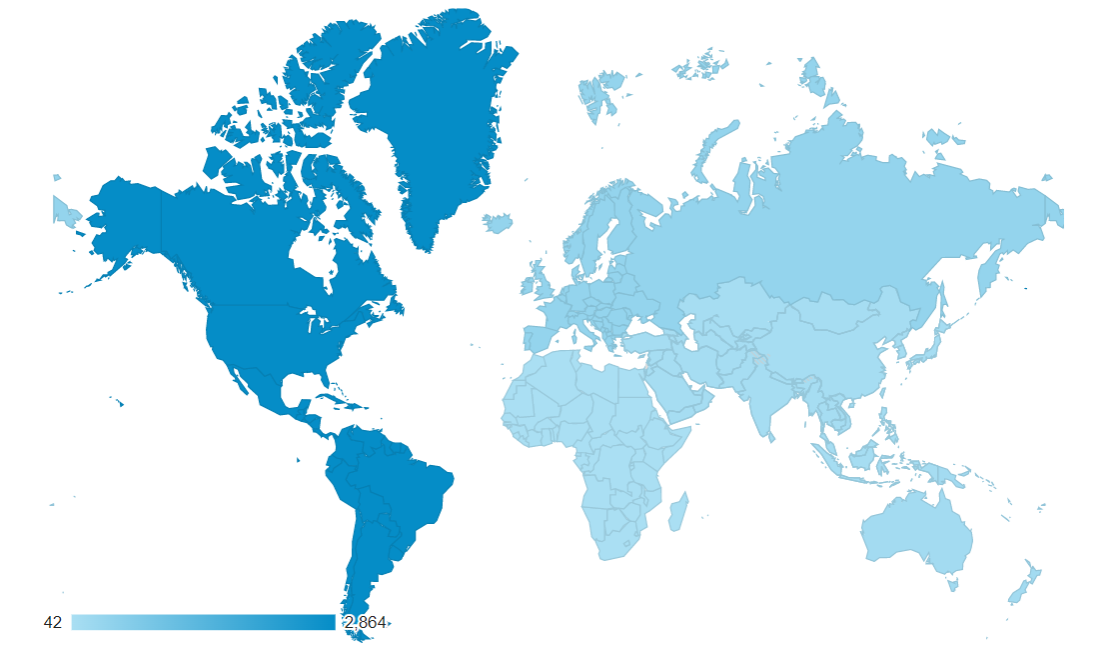
Geographical location of users by country.

During the study period, from April 8, 2020, to May 3, 2020, we had a total of 3588 active users of the web application, of which 3349 users (93.3%) had a single session and 239 users (6.7%) had multiple sessions (Table 3). According to the ISO 3166 country code, the web application was used in 82 countries (Figure 5). The 3588 users were located in the Americas (2864/3588, 79.8%), Europe (411/3588, 11.5%), Oceania (166/3588, 4.6%), Asia (103/3588, 2.9%), and Africa (42/3588, 1.2%).

Of the 3588 users, 3561 (93.3%) were new visitors, so they logged in only 1 time during the study period. Users who returned to the web application (“returning visitors”) numbered 254 (6.7%). This represents 3924 sessions, including 3349 (85.3%) single and 575 (14.7%) multiple sessions. Of these, the majority (1261/3924, 32.1%) of the sessions varied in duration from 181 seconds to 600 seconds, representing an average time spent on the web application of 3.18 minutes.

**Table 3:** Users and use patterns (n=3588).

Use patterns				Results
**Active users n (%)**				
	Single session users		3349 (93.3)	
	Multisession users		239 (6.7)	
	Survey respondents		182 (5.1)	
**Geographic characteristics^a^, n (%)**				
	**Language**			
		English	3175 (88.5)	
		French	199 (5.5)	
		Both	1 (0.1)	
		Other	213 (5.9)	
	**Location**			
		United States	1830 (51.0)	
		Canada	947 (26.4)	
		Australia	149 (4.2)	
		United Kingdom	108 (3.0)	
		Other	554 (15.4)	
	**Location by continent, n (%)**			
		Americas	2864 (79.8)	
		Europe	411 (11.5)	
		Oceania	166 (4.6)	
		Asia	103 (2.9)	
		Africa	42 (1.2)	
**Technology, n (%)**				
	**Device category**			
		Desktop	1745 (48.7)	
		Cell phone	1692 (47.1)	
		Tablet	152 (4.2)	
	**Browser**			
		Chrome	1677 (46.7)	
		Safari	1184 (33.0)	
		Safari (in-app)	230 (6.4)	
		Firefox	206 (5.7)	
		Android WebView	150 (4.2)	
		Samsung Internet	71 (2.0)	
		Edge	29 (0.8)	
		Opera	20 (0.6)	
		Mozilla Compatible Agent	19 (0.5)	
		Amazon Silk	2 (0.1)	
	**Operating system**			
		iOS	1145 (31.9)	
		Windows	899 (25.1)	
		Macintosh	788 (21.9)	
		Android	697 (19.4)	
		Chrome OS	37 (1.0)	
		Linux	21 (0.6)	
		BlackBerry	2 (0.1)	
**Behavior**				
	New visitors, n (%)		3561 (93.3)	
	Returning visitors, n (%)		254 (6.7)	
	**Count of sessions^b^, n (%)**			
		Single session users	3349 (85.3)	
		Multisession users (2 to 10 sessions)	575 (14.7)	
	**Session duration, (number of sessions)**			
		0-10 seconds	1217	
		11-30 seconds	220	
		31-60 seconds	205	
		61-180 seconds	790	
		181-600 seconds	1261	
		601-1800 seconds	224	
		≥1801 seconds	7	
	**Session duration (minutes:seconds), mean**			
		All users	00:03:18	
		Single session users	00:03:27	
		Multisession users	00:02:23	
		New visitors	00:03:26	
		Returning visitors	00:01:59	

^a^n=3588.

^b^3924 sessions by 3588 users.

In the behavioral flow for this web application, users have 5 points of interaction: landing page, home page, avatar creation, animation, and extra information with the optional survey (Figure 1). From the initial point of interaction (the landing page), of the 3924 sessions, 3690 (94%) sessions of total traffic arrived at the home page. From there, of the 3924 sessions, 3110 (79.2%) reached the avatar creation page, 2148 (54.7%) reached the animation page, and 681 (17.4%) reached the extra information with the optional survey page, meaning they had to have viewed the animation and fully completed the user's journey.

These data enabled us to obtain information from participants' usage patterns, the journey through the web application, and to draw a user profile. The typical user had a browser set in English (3175/3588, 88.5%), was in the United States (1830/3588, 51.0%), and was mainly exposed to animation on desktop (1745/3588, 48.7%) from Chrome (1677/3588, 46.7%) or cell phone (1692/3588, 47.1%) with iOS (1145/3588, 31.9%) as the primary operating system. This new user logged in for a single session of at least one time for an average of 181 seconds, or an average of 3:26 minutes, and did not complete the optional survey on distancing.

## Discussion

### Principal Findings

The aim of this study was to understand the impact of a dynamic visualization on distancing during the early months of the COVID-19 pandemic. Our results led us to 3 principal observations, which we detail in the following paragraphs.

First, given that the large majority of survey participants were already practicing distancing, we suggest that such communication materials might have dual roles. The first role is the more obvious one of helping to prompt behavior change. In our study, among the small subsample of survey respondents who reported not already practicing distancing, 29% indicated an intention to increase their practice of distancing in the coming week. Due to low numbers, this was not statistically significant. The second role may be to help reinforce existing desired behaviors. A substantial number of people went through the application in its entirety and responded to the survey at the end, indicating that they were already practicing distancing. Our survey did not include questions about why they might have spent this time, so we are unable to draw conclusions, but we hypothesize that, especially in the context of the early stages of a pandemic, people may seek reassurance that they are doing the right things. Public health communications may therefore offer information that is helpful in more subtle ways than prompting immediate behavior change. In other words, there may be a place for communications that are explicitly designed to reinforce existing behaviors and to be shared or shown to others, so that individuals can use them in their discussions with family, friends, and colleagues. Future studies should consider further exploring such sharing intentions.

Second, we observe that characteristics such as gender and country of residence may influence the impact of digital public health communications. Our web application received 3588 unique hits from 82 countries after 26 days, having been shared on social media across Canada. The majority of survey respondents reported higher education levels. This may suggest that the application itself reached more people with higher levels of education, or it may reflect higher willingness to complete an optional survey among people with higher levels of education. We also noted that gender and place of residence interacted statistically significantly with distancing intentions. Specifically, among those living outside of Canada, participants who identified as women appeared to be impacted toward prosocial behavior (ie, distancing to protect oneself and others) following exposure to our intervention, while those who identified as men appeared to be impacted in an opposite direction.

Third, usage analytics revealed that most people who abandoned the application gave up at the point at which they were asked to create 9 custom avatars prior to viewing the animation integrating those avatars. The rationale of custom avatars was to highlight the impact of distancing on people known to the user. However, creating 9 avatars may have been too much to ask. This suggests that, for future such applications, it will be important to find the right balance between personalization and the amount of information and time asked of the user.

### Comparison With Previous Literature

As recommended by Gough and colleagues [36], we had some success disseminating our message using Twitter. Although Twitter has since undergone changes and may no longer function in quite the same way as it did in 2020, social media remains an important vehicle for reaching a wide range of people. Indeed, during the COVID-19 pandemic, public health experts increasingly used social media to disseminate messages directly [2]. To optimize the impact of evidence-based messages, academic and other institutional dissemination efforts may benefit from consultation, advice, or collaboration with social media experts.

Our findings around potential differences in how people with varying identity characteristics received our intervention align with other studies showing that such characteristics matter and must be considered when designing public health messages [2,13,14,37-42]. As outlined by the World Health Organization in its strategy to combat COVID-19, triggering the right action requires delivering the right message to the right audience at the right time [43].

This research built on previous work by Hakim and colleagues [19,44] developing and evaluating an interactive visualization about how herd immunity or community immunity works. By incorporating avatars and considering the role of emotions, Hakim and colleagues [19] aimed to personalize communication about infectious disease transmission. Our web application offers an adaptation of the original application, suggesting that such a visualization’s core functionality may be usable across related contexts [45]. For example, although distancing is no longer the recommended avenue for reducing COVID-19 transmission, the visualization may be adapted to demonstrate the impact of mask wearing or vaccination on transmission. By linking our web application with Google Analytics, we obtained indirect feedback about user experience, technology usage, and most importantly, user pain points, enabling us and other teams building similar applications to better balance user burden and intervention effectiveness. Put plainly, a digital public health intervention is unlikely to be effective if people abandon it halfway through. According to our site usage metrics, we had 2 main throttle points: avatar creation and animation viewing. The avatar creation process (selecting an avatar, then customizing skin color, hair color, glasses, facial hair, and head coverings like hijabs, turbans, and caps) can take a long time, especially for new users. To address this issue, in future iterations building on this application, we will offer an option to automatically create additional avatars. Although no interaction was required in the animation viewing process, people might have abandoned it because it was too long, they didn’t wish to spend the time, they lacked the bandwidth to stream the video, or other reasons. Currently, we are refactoring this process to make it easier for future users. As a solution to this issue, we added the ability to move the visualization forward or backward and aim to use shorter animations.

### Limitations

Our study had 4 main limitations. First, the nonrandomized study design limited our ability to draw firm conclusions about the actual effect of our web application on distancing. Second, the sample of survey respondents was smaller than the population of application users, with a total of 182 responses to the optional survey out of 3588 unique hits. In addition, given our recruitment methods and high baseline distancing practices, we likely observed selection bias in survey respondents. Third, we were able to measure distancing intentions but unable to measure self-reported behavior in a follow-up survey. Fourth and finally, the Google Analytics data limited our analyses. Such data are structured primarily for the needs of those analyzing marketing metrics such as acquisition, behavior, and conversion, not for scientific analyses. In future studies, we aim to explicitly link survey data with usage metrics for each individual study participant.

### Strengths

This study has 3 main strengths. First, using agile development, we demonstrated the ability to adapt an existing application about herd immunity or community immunity, already optimized for users’ cognitive and emotional responses, to a new infectious disease context. This reinforces potential avenues for further research using already available software and approaches. Second, using social media showed that we could disseminate this type of material rapidly and reach a larger sample of the population than the convenience sample we had originally defined. Third and finally, this research provided insight on user experience to inform future applications designed to help members of the public understand the science of infectious disease transmission and how epidemiological evidence applies to them as individuals and to those around them.

### Conclusions

We developed a web application to help people understand the relationship between individual-level behavior and population-level effects in the context of infectious disease spread. This paper suggests that personalized risk communication, such as personalized avatar-based visualizations, could be effective. This study also demonstrates how agile development can be used to quickly create personalized risk messages for public health issues like a pandemic. The nonrandomized design of this rapid study does not allow conclusions about the web application’s effectiveness on distancing or knowledge retention. Future work may improve the visualization and involve more user testing.

## Appendix

Multimedia Appendix 1 Storyboard and script.

Multimedia Appendix 2 Data set that was generated and analyzed.

## Data Availability

All data generated or analyzed during this study are included in this published article and are available in Multimedia Appendix 2.
