# Supplementary material for: A Personalized Avatar-Based Web Application to Help People Understand How Social Distancing Can Reduce the Spread of COVID-19: Cross-sectional, Observational, Pre-Post Study
Source: JMIR Form Res. 2023 Apr 25;7:e38430. doi: 10.2196/38430 (PMC10170367; doi:10.2196/38430)
Supplement: Multimedia Appendix 1 [file formative_v7i1e38430_app1.docx]

## **Appendix 1. Storyboard and Script**

|  | **English narration** | **Narration française** |
| --- | --- | --- |
| Creation of 9 avatars  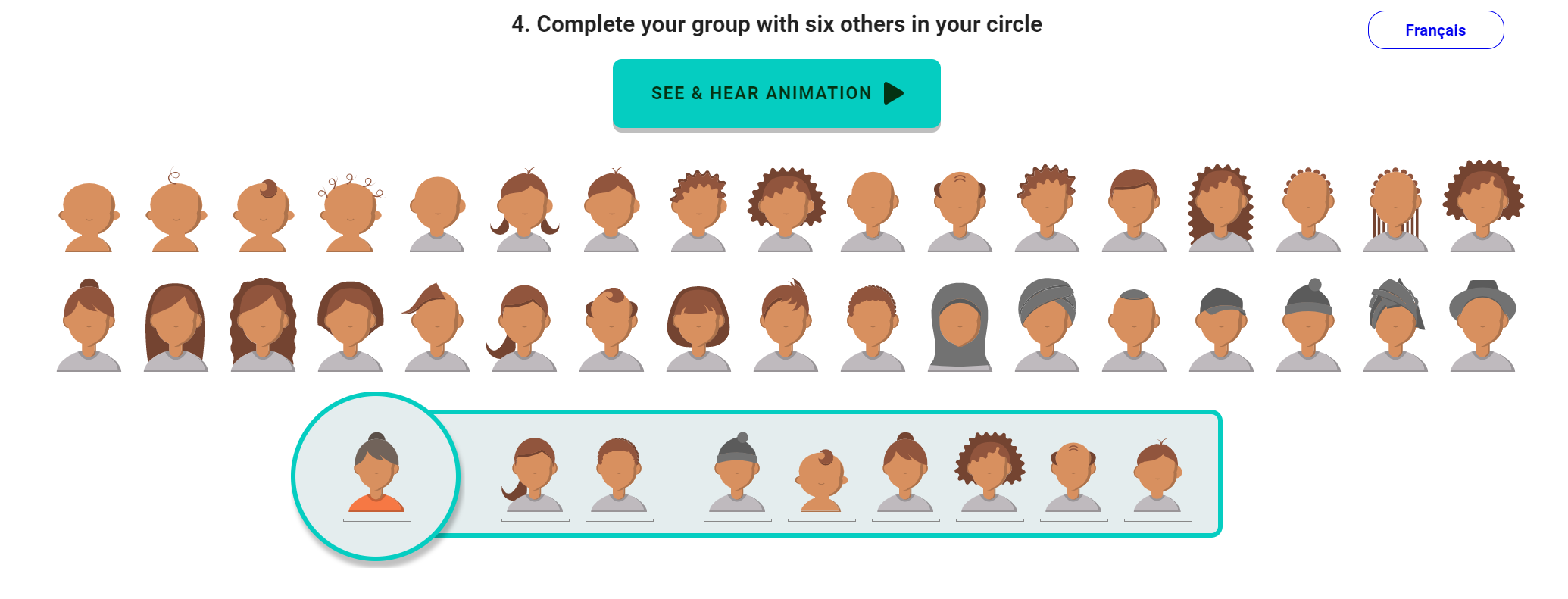  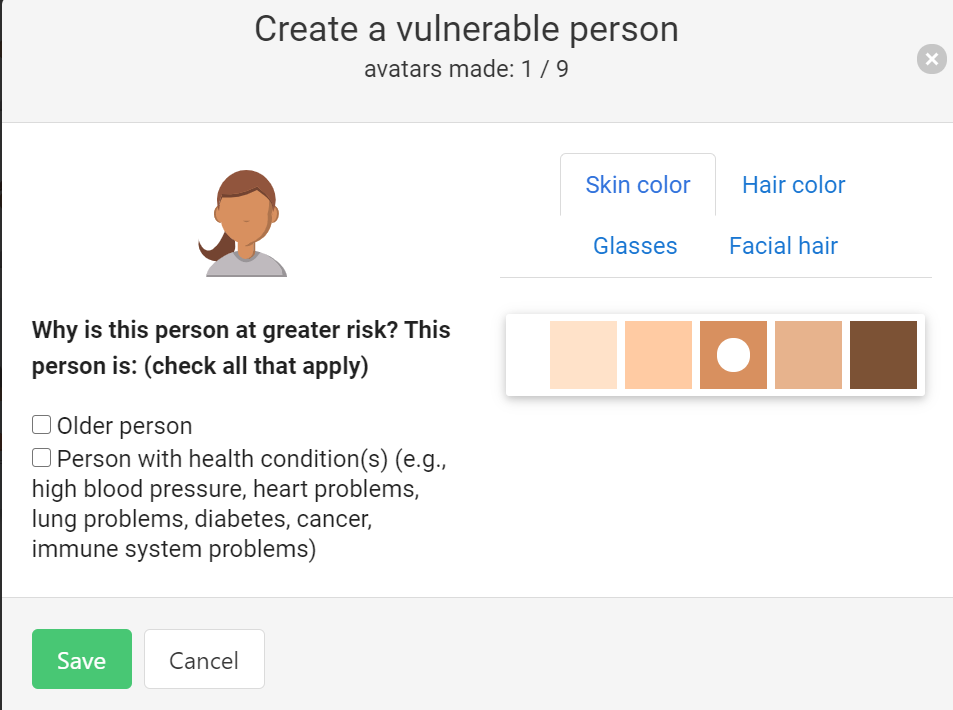 | n/a | n/a |
| [Introduction]  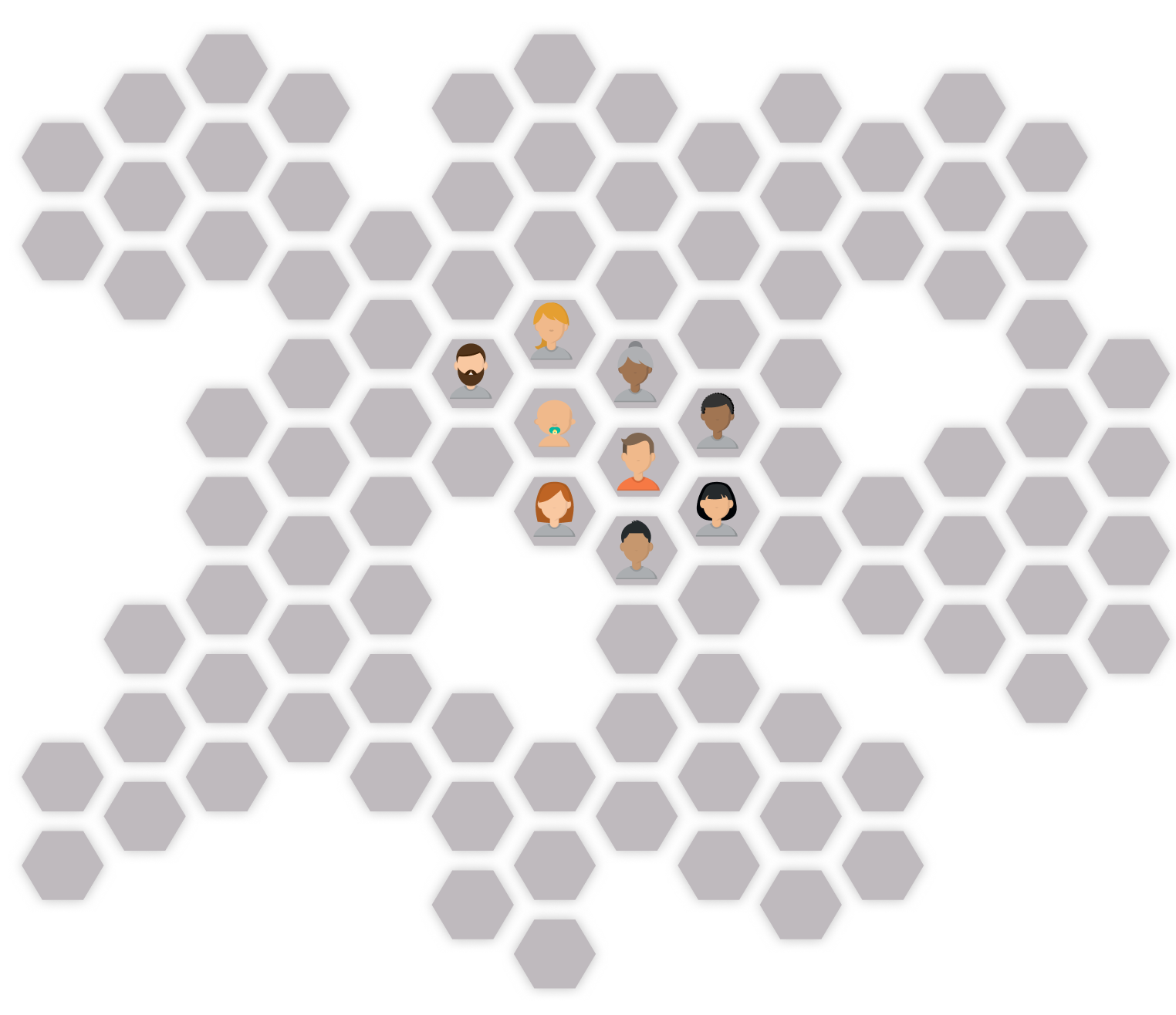 | What role does each of us play in protecting our community?  [descriptive text for blind or visually impaired: The image shows 100 grey hexagons, each representing one person, arranged on the screen.] | Quel rôle jouons-nous dans la protection de la communauté?  [Texte descriptif pour aveugles ou malvoyants: Une image présente 100 hexagones gris, chacun représentant une personne.] |
| Community | | |
| 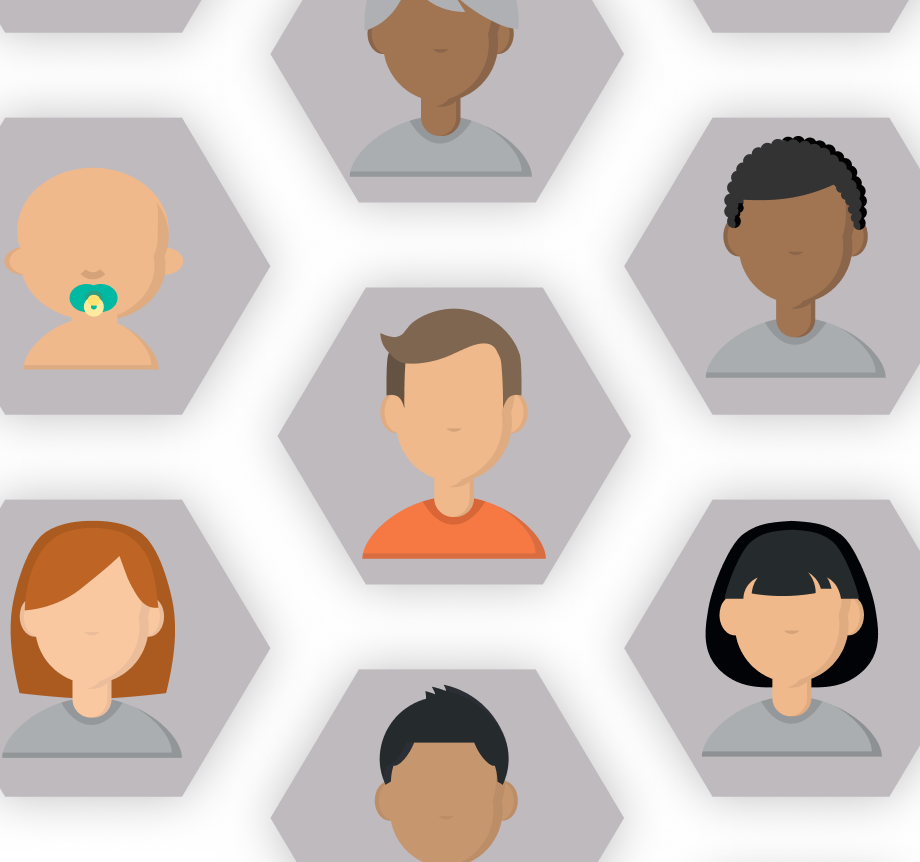 | Imagine this is you.  [The video zooms in on the hexagon in the middle of the 100. The hexagon has an avatar representing you, wearing an orange T-shirt.] | Imaginez que c’est vous  [La vidéo zoome sur l’hexagone au milieu des 100. Cet hexagone comporte un avatar portant un t-shirt orange. L’avatar vous représente.] |
| 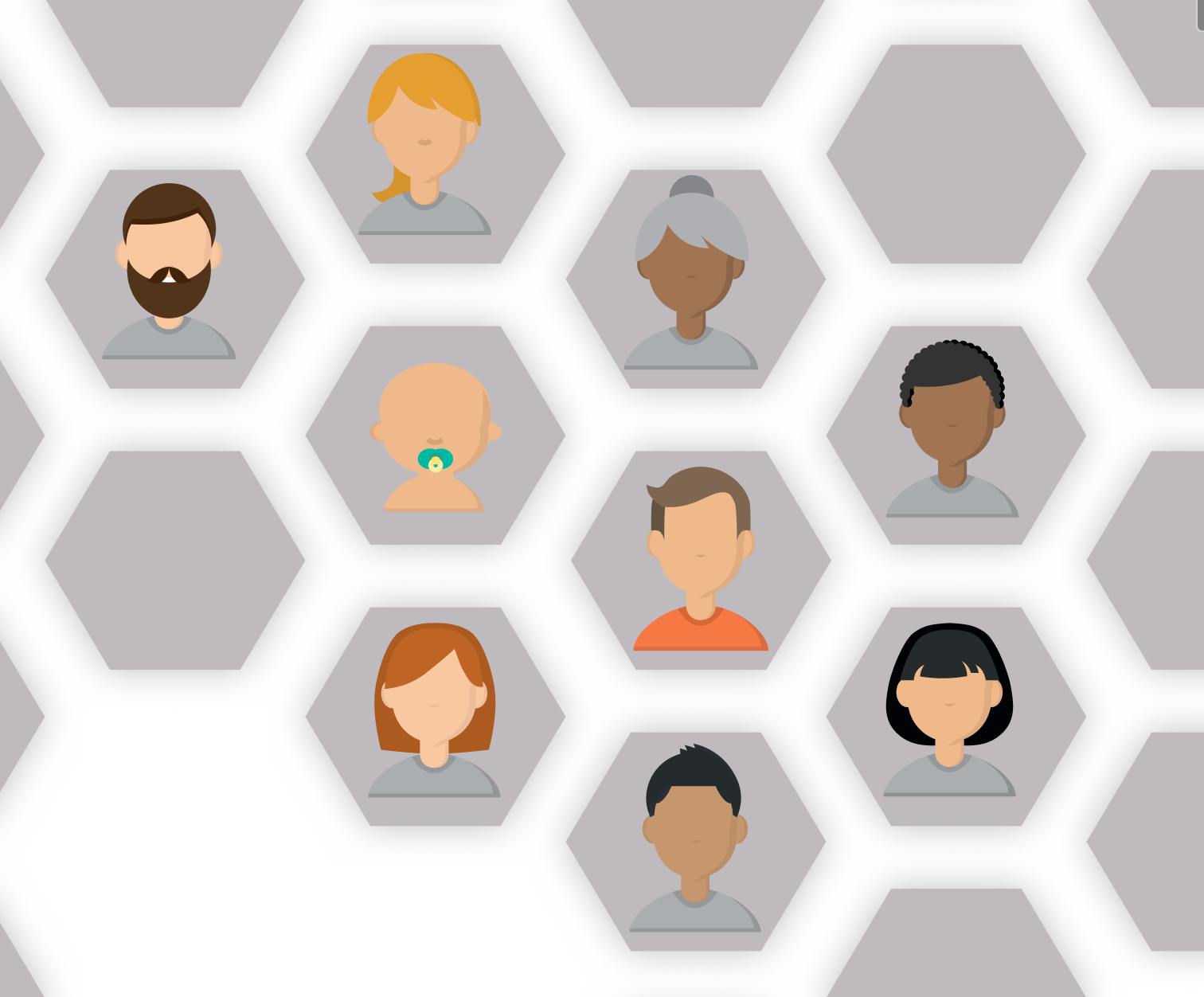 | These are people around you—people you see often, like your family, friends, neighbours, or coworkers, and the people around *them* that *they* see often.  [The video zooms out a little to show other avatars and hexagons around you. The other avatars are wearing dark grey T-shirts.] | Voici des personnes que vous côtoyez souvent, comme votre famille, vos amis, voisins, ou collègues, et les personnes qu’elles-mêmes côtoient  souvent.  [Le zoom de la vidéo s’éloigne un peu et montre les autres avatars qui vous entourent. Ceux-ci portent des t-shirts gris foncé.] |
| 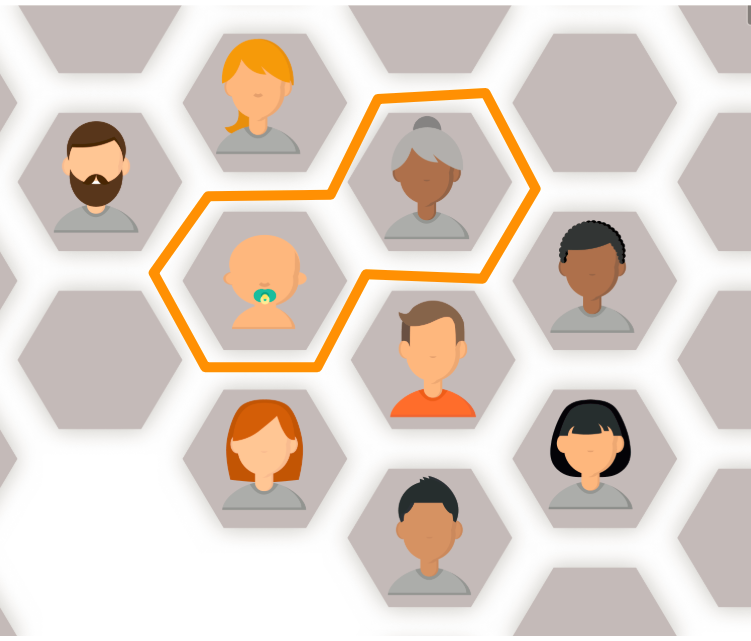 | Some of these people, like older people, or those with health problems (for example, high blood pressure, other heart problems, lung problems, diabetes, cancer, or immune system problems) …  [Orange lines appear around two hexagons next to you. These hexagons have avatars representing vulnerable people close to you.] | Parmi ces personnes, certaines sont des personnes âgées, d’autres ont des problèmes de santé (comme de l’hypertension et d’autres problèmes de cœur, des problèmes aux poumons, un diabète, un cancer, ou un affaiblissement du système immunitaire.)  [Des lignes oranges apparaissent autour de deux hexagones à côté du vôtre. Ces hexagones contiennent des avatars qui représentent des personnes vulnérables de votre entourage.] |
| 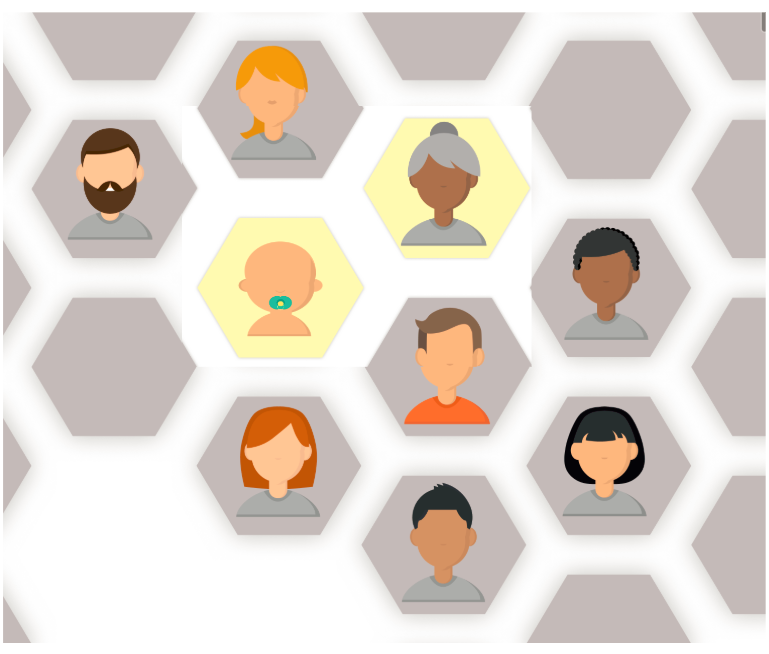 | … are more vulnerable to contagious diseases like the Coronavirus. This is because it can make them sicker and they may be at higher risk of dying of it.  [The background of the two hexagons representing vulnerable people changes from grey to pale yellow.] | Ces personnes sont considérées comme étant plus vulnérables aux maladies comme le Coronavirus, parce que ça pourrait les rendre plus malades et elles auraient un risque plus élevé d’en mourir.  [La couleur des deux hexagones représentant des personnes vulnérables change et passe de grise à jaune pâle.] |
| Infection/Disease | | |
| 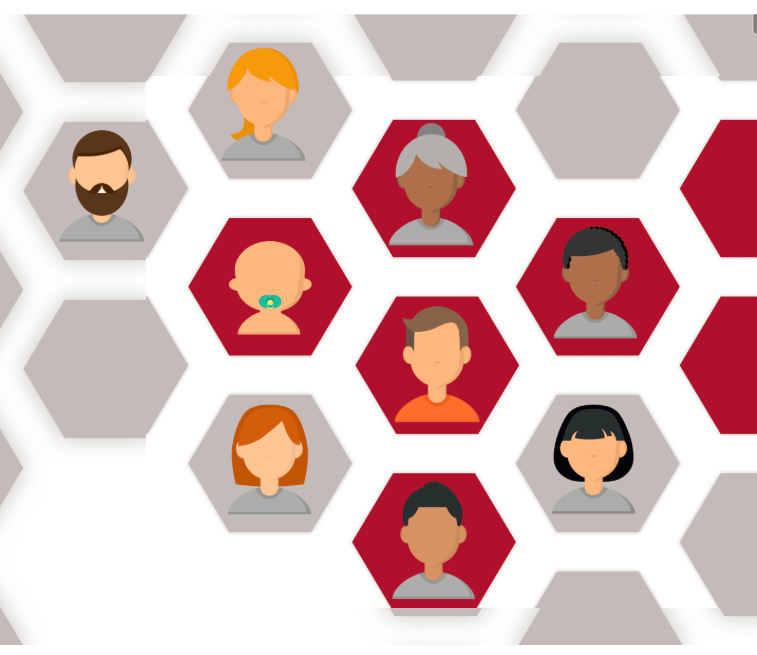 | When a contagious disease enters your community, anyone can catch it, pass it on to those around them, get sick, or even die from it.  [A red line enters the close-up of hexagons shown on the screen. It touches a grey hexagon. The hexagon turns red. The T-shirt of the avatar also turns red. Then a line extends from that red hexagon to another grey hexagon, it turns red, and so on. Soon, about half of the hexagons have been touched by a red line and have turned red, including your avatar and the two avatars representing vulnerable people.] | Lorsqu’une maladie contagieuse comme le Coronavirus se propage dans une communauté, n’importe qui peut l’attraper, la transmettre, tomber malade ou même en mourir.  [Une ligne rouge pénètre dans le gros plan des hexagones affichés à l’écran. Elle touche un hexagone gris qui devient rouge ainsi que le T-shirt de l’avatar. Puis, la ligne s’étend de cet hexagone rouge à un autre hexagone gris, qui devient rouge à son tour, ainsi de suite. Bientôt, environ la moitié des hexagones ont été touchés par une ligne et sont devenus rouges, y compris votre avatar et les deux avatars représentant les personnes vulnérables. |
| Immunity | | |
| 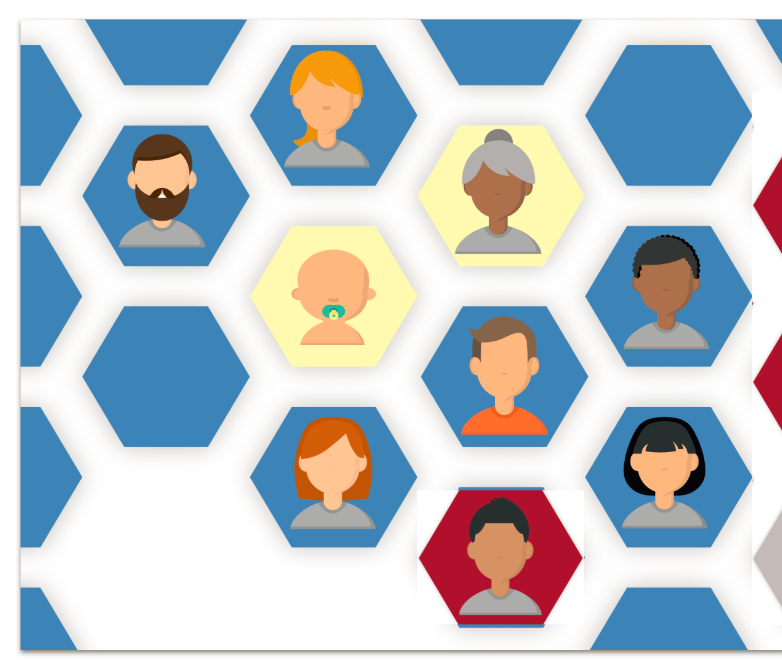 | When people are *immune* to a contagious disease, shown here by blue hexagons, they are less likely to catch it, pass it on, get sick, or die from it. This is because their immune system, the body’s protection system, recognizes the disease and knows how to fight it.  [Many hexagons’ background turns blue to represent having been vaccinated. The red hexagons extend lines to the blue hexagons, but unlike the grey hexagons, the blue ones don’t turn red upon contact by a red line.] | Quand les personnes sont *immunisées* contre une maladie contagieuse, représentés ici par des hexagones bleus, elles sont moins susceptibles d’attraper la maladie, de la transmettre, en tomber malade ou en mourir. C’est dû au fait que leur système immunitaire, le système de protection du corps humain, reconnaît la maladie et sait comment la combattre.  [Plusieurs fonds d’hexagone deviennent bleus pour représenter le fait d’être vacciné. Les hexagones rouges étendent des lignes aux hexagones bleus, mais contrairement aux gris, ils ne deviennent pas rouges au contact d’une ligne rouge.] |
| Social Distancing | | |
| 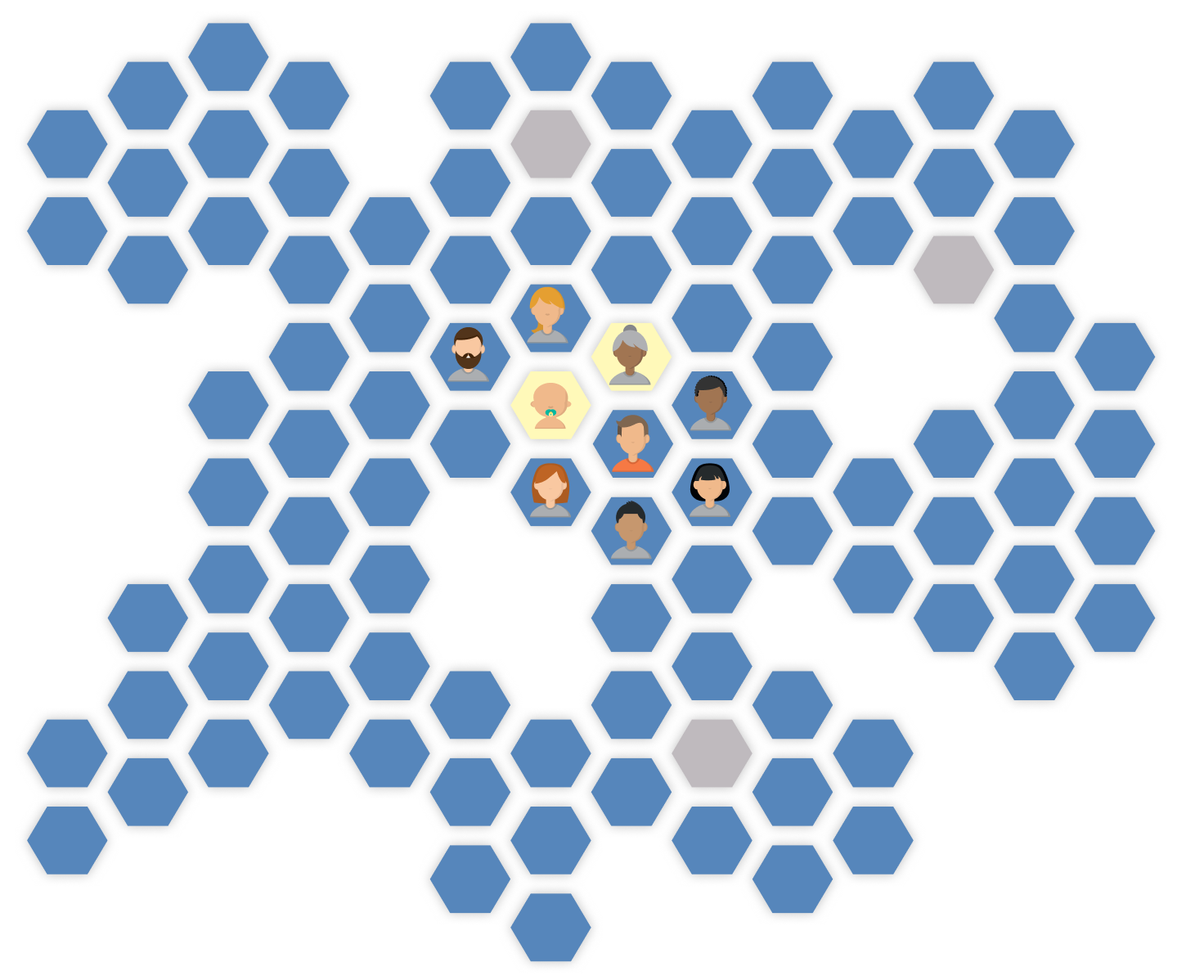 | When enough people are immune to a contagious disease, it creates a protective barrier…  [The video zooms out and shows all 100 hexagons, the majority of which are blue.] | Quand il y a suffisamment de personnes immunisées contre une maladie contagieuse, ça crée une barrière protectrice autour de toute la communauté.  [La caméra recule et montre les 100 hexagones dont la majorité est bleue.] |
| 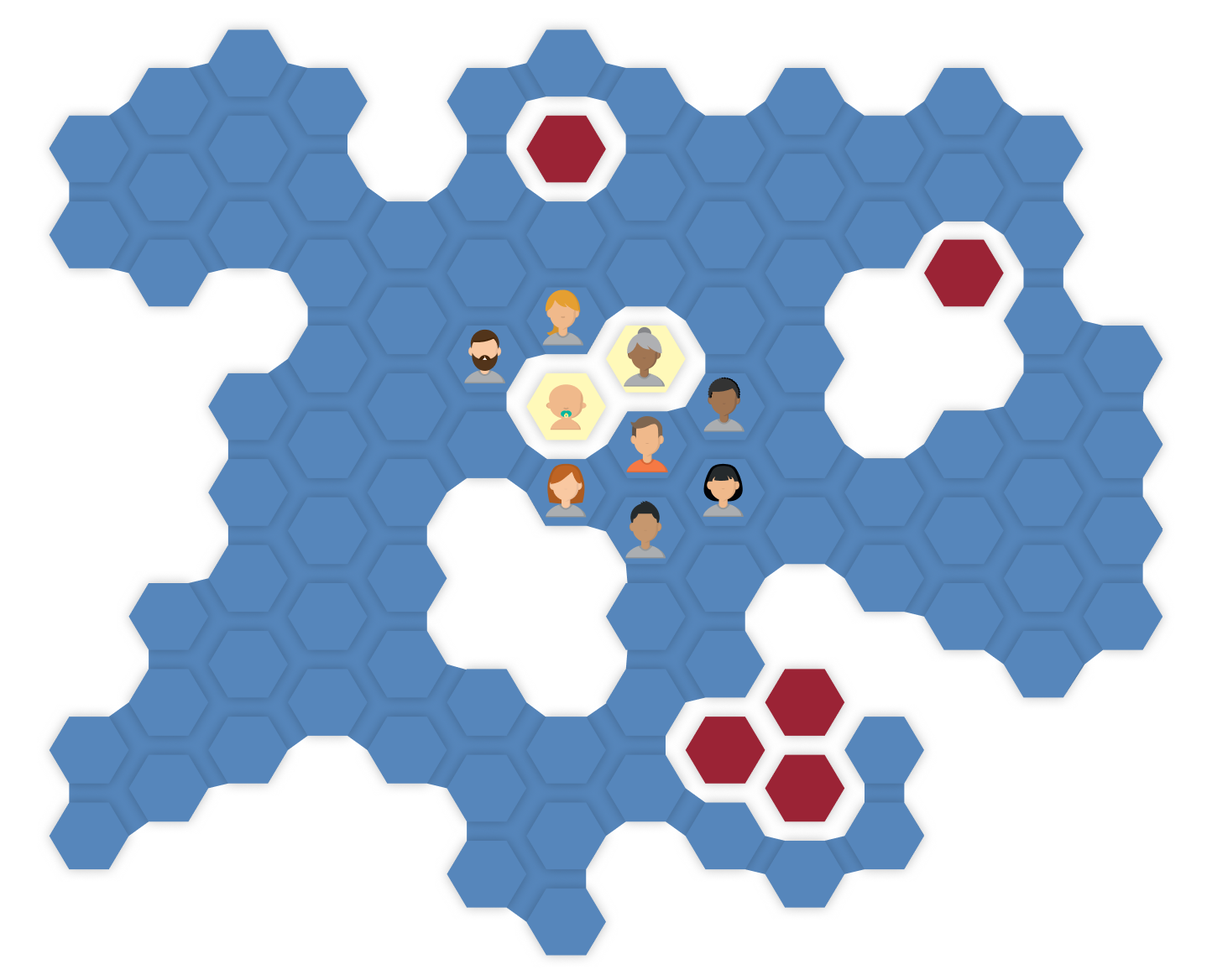 | … called “herd immunity” or “community immunity” that helps stop contagious diseases from spreading from one person to another in a community.  [The space in between the blue hexagons fills in with a paler blue, and the whole connected shape pulses gently. The two hexagons representing vulnerable people are still yellow and are surrounded by the blue shape.] | Cette barrière se nomme « l’immunité de groupe” »ou “l« immunité collective”. »’immunité collective empêche la maladie contagieuse de se propager d’une personne à l’autre.  [L’espace entre les hexagones bleus se remplit d’un bleu plus clair et toute la forme connectée clignote doucement. Les deux hexagones représentant les personnes vulnérables sont toujours jaunes, et sont entourés par la forme bleue.] |
| 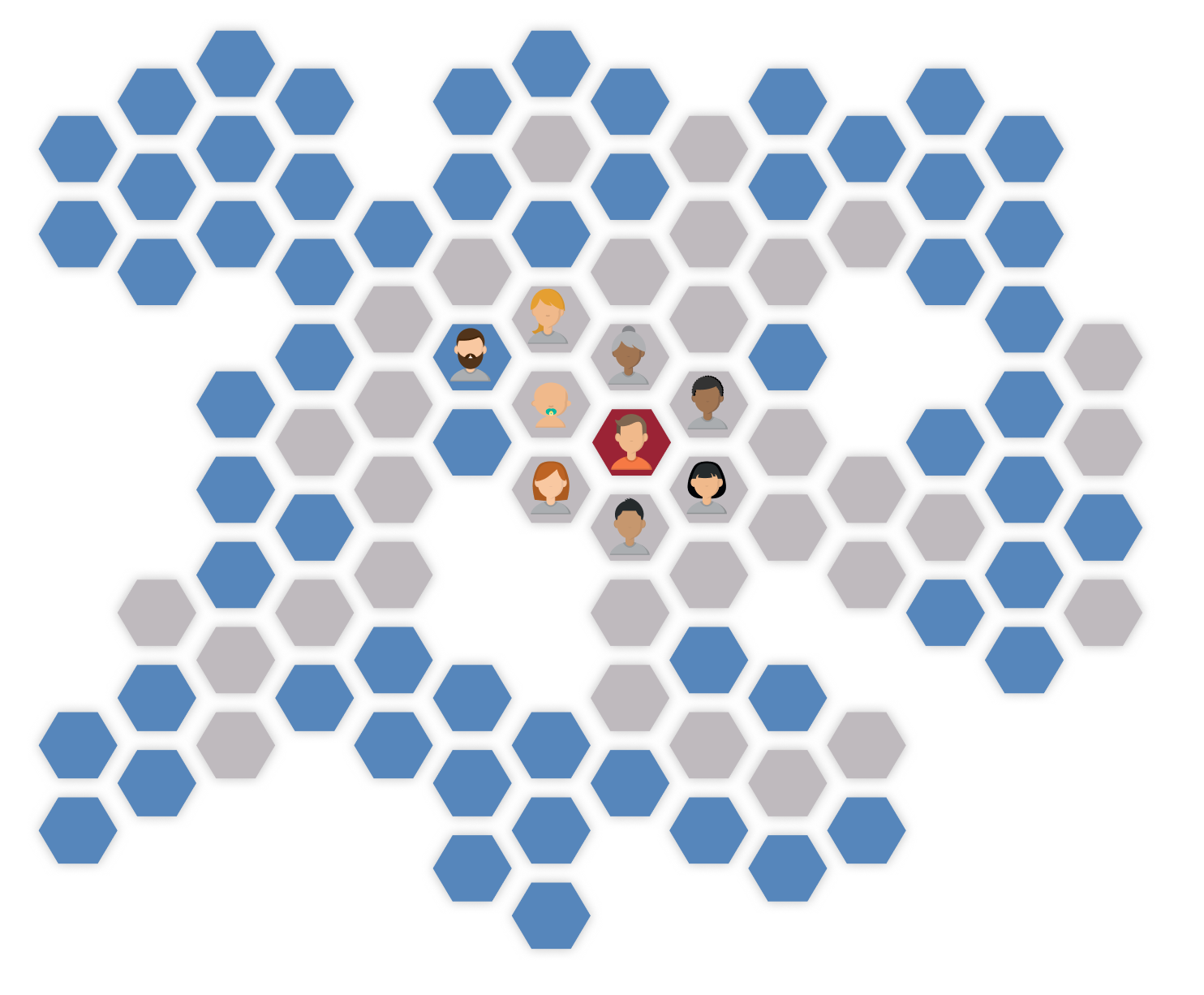 | But the Coronavirus is a new virus, so we don’t have immunity yet. Scientists are working hard on vaccines and treatments, but this work takes time. In the meantime, we are all at risk of being exposed to the virus, catching it, and passing it on to others, so we have to use different ways to protect our communities.  [The blue colour in the hexagons fades. All are grey again.] | Le Coronavirus étant un nouveau virus, nous n’avons pas encore d’immunité. Les scientifiques travaillent ensemble pour élaborer des vaccins et des traitements, mais leurs efforts prendront du temps. En attendant, nous pouvons tous être exposés et courir le risque d’être contaminé et de transmettre le virus. Nous devons donc utiliser différents moyens pour protéger nos communautés.  [Le bleu des hexagones s’estompe et ils deviennent gris.] |
| Proposed version / version proposée:  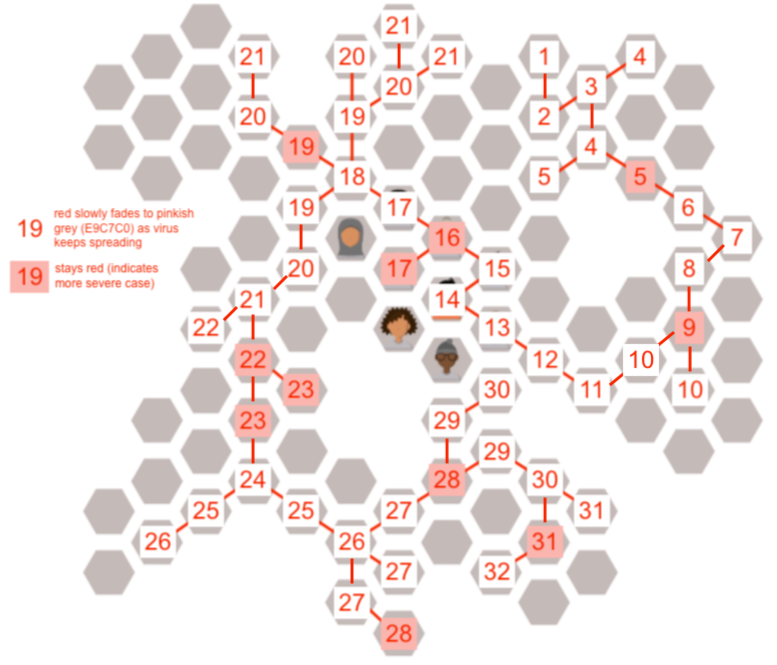  (see Fig 1 below for final version after discussions with infectious disease modelers) | If we don’t do anything, the Coronavirus can easily spread through your community. It can reach you, the people around you, and others in your community. Some people will have a milder case, perhaps even without symptoms. Milder cases are shown by hexagons that turn red, then fade to pink. But other people, the hexagons that stay red, will have severe cases and may need to go to the hospital. If too many people have severe cases at once, hospitals may not have enough beds, equipment, or staff to care for everyone.  This could be a problem for anyone who needs urgent medical attention, whether for the Coronavirus or other reasons; for example, a heart attack or car accident.  [A red line enters the 100 hexagons and spreads from each hexagon to 1, 2, or 3 others, spreading around the group. When it stops, 51 hexagons have been coloured red. 41 have faded to pinkish-grey while 10 remain dark red.] | Si nous ne faisons rien, le Coronavirus peut facilement se répandre dans votre communauté. Il peut vous atteindre vous, ainsi que les gens autour de vous et d’autres personnes qui vous sont chères. Pour certaines personnes, il s’agira d’une maladie assez bénigne, peut-être même sans aucun symptôme. Les cas plus légers sont représentés par des hexagones qui deviennent rouges, puis pâlissent en rose. Pour d’autres personnes, les hexagones qui demeurent rouges, la maladie sera plus grave et elles devront peut-être se rendre à l’hôpital. Si un trop grand nombre de personnes se retrouvent dans un état grave en même temps, les hôpitaux n’auront peut-être pas assez de lits, d’équipement médical ou de personnel pour toutes les soigner.  Cela pourrait être un problème pour toute personne atteinte d’un cas grave de Coronavirus, et aussi pour toute personne ayant un besoin urgent de soins médicaux pour d’autres raisons, par exemple une crise cardiaque ou un accident de voiture.  [Une ligne rouge entre au milieu des 100 hexagones et se propage à 1, 2, ou 3 autres et s’étend au groupe. Quand elle s’arrête, 51 hexagones ont été colorés de rouge. 41 ont pâlis en un gris rosé tandis que 10 restent rouge foncé.] |
| 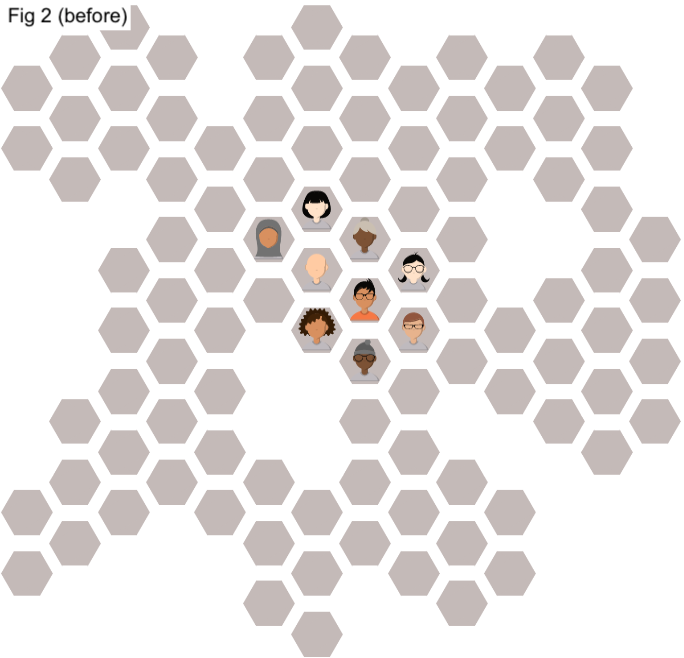  becomes / devient  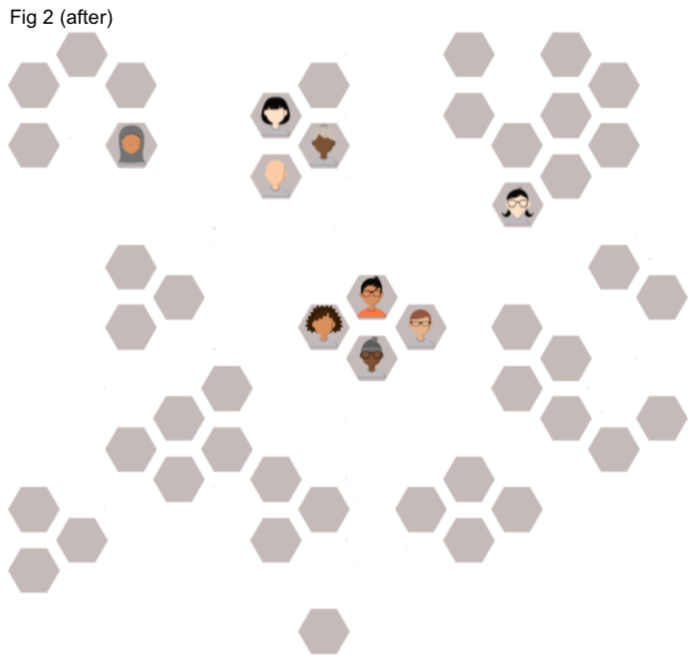 | Your community can help keep the Coronavirus from spreading by reducing contact between people. This is called “distancing.” Some are calling it “social distancing,” while others are calling it “physical distancing.”  [Many of the hexagons disappear and those remaining are now in small groups ranging in size from 1 to 9 hexagons, spread out from each other.] | Votre communauté peut contribuer à empêcher la propagation du Coronavirus en réduisant les contacts entre les personnes. C’est ce qu’on appelle la « distanciation" » Certains parlent de "d« stanciation sociale", »andis que d’autres parlent de "dis« anciation physique".  [Plusieurs des hexagones disparaissent et ceux qui restent sont désormais en petits groupes variant d’une taille de 1 à 9 hexagones séparés les uns des autres.] |
| 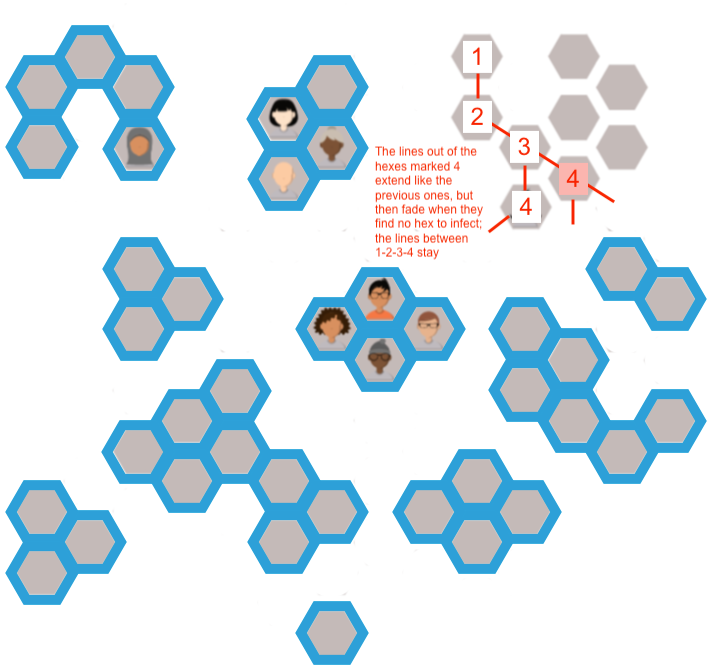 | Whatever you call it, it means the same thing: stay home, don’t have any visitors over, and keep your distance from others in public. When you have to go out (for example, to get food, or take a walk outside) stay 2 metres (or about 6 ft) away from other people so that droplets containing the virus can’t get from one person to another. This helps keep the virus from spreading in your community.  [One grey hexagon turns red and spreads the red colour to four other hexagons, to make five red hexagons. Four of the red hexagons fade to pinkish-grey. One stays red. All the other separate groups of hexagons become surrounded by blue borders. The red lines representing the virus spreading cannot reach from the red hexagons to the groups surrounded by blue.] | Quel que soit le nom utilisé, il veut dire la même chose: restez chez vous, ne recevez pas de visiteurs et tenez-vous à distance des autres dans les lieux publics. Lorsque vous devez sortir (par exemple, pour chercher des provisions ou marcher dehors), restez à 2 mètres (environ 6 pieds) des autres personnes afin que les gouttelettes contenant le virus ne puissent pas passer d’une personne à l’autre. Cela permet d’éviter que le virus ne se propage dans votre communauté.  [Un hexagone gris devient rouge et transmet la couleur rouge à quatre autres hexagones. Quatre des hexagones rouges pâlissent en un gris rosé. Un reste rouge. Tous les autres groupes séparés d’hexagones se retrouvent entourés de bordures bleues. Les lignes rouges représentant la propagation du virus ne peuvent pas partir des hexagones rouges pour atteindre les groupes d’hexagones entourés de bleu.] |
| Conclusion | | |
| 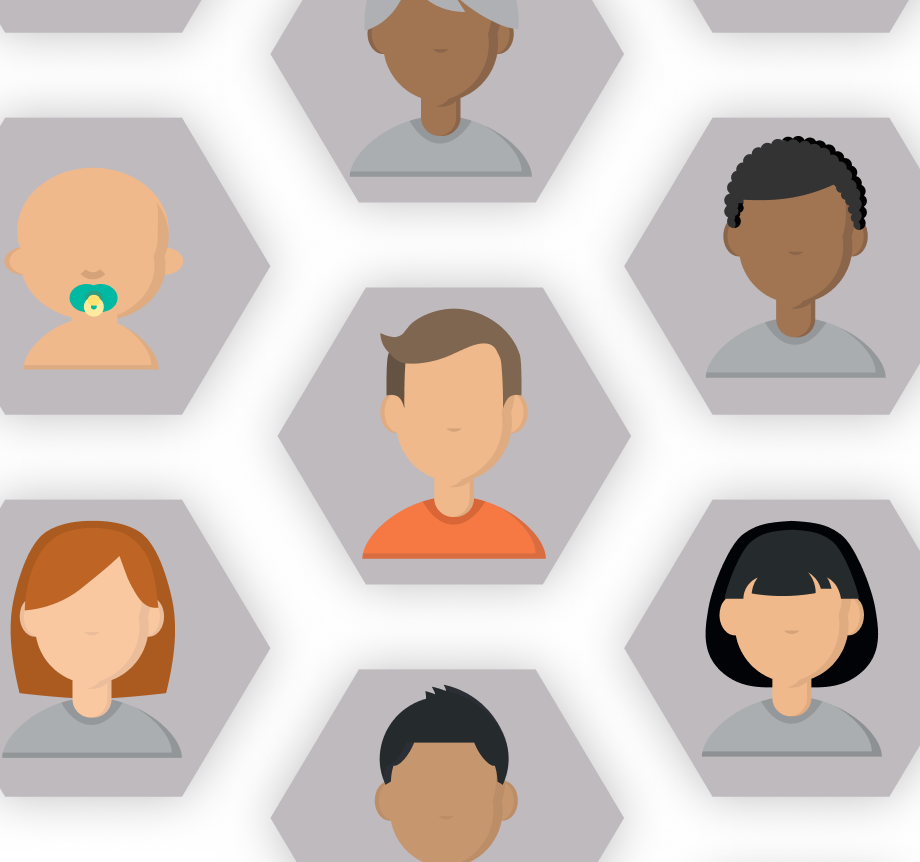 | Your actions right now affect you—and other people. By practising distancing, you are helping to keep the Coronavirus from spreading. This protects everyone in your community. Thank you for doing your part.  [Video zooms back in to focus on the hexagon and avatar representing you.] | Vos actions actuelles vous affectent vous, ainsi que d’autres personnes. En pratiquant la distanciation, vous contribuez à empêcher le Coronavirus de se propager. Vous protégez tous les membres de votre communauté. Merci de contribuer à l’effort collectif.  [La vidéo fait un zoom avant pour se concentrer sur l’hexagone et l’avatar qui vous représentent] |
|  | TEXT (not narration)   Thank you for watching this animated video. Here is more information from the Public Health Agency of Canada about [distancing and how to prevent the Coronavirus](https://www.canada.ca/en/public-health/services/diseases/2019-novel-coronavirus-infection/prevention-risks.html#p).  SUMMARY  Distancing (“physical distancing” or “social distancing”) means:  —Stay home.— Don’t visit anyone else.  —Don’t have any visitors over.— When you must go out, stay 2 metres (6 feet) away from others.  Also—- Wash your hands often with soap and water for 20 seconds.  People who are sick or who have returned from travel need to do more than distancing. For more information: <https://www.canada.ca/en/public-health/services/publications/diseases-conditions/self-monitoring-self-isolation-isolation-for-covid-19.html>  We would really appreciate your answers to a few questions. Click here for a short (5 questions) survey about this video. | TEXTE (pas de narration)  Merci d’avoir regardé cette vidéo animée. Voici plus d’informations de l’Agence de santé publique du Canada sur [la prévention du Coronavirus](https://www.canada.ca/fr/sante-publique/services/maladies/2019-nouveau-coronavirus/prevention-risques.html#p).  EN RESUME  La distanciation (distanciation physique ou distanciation sociale) signifie:   - Rester à la maison. - Ne pas faire de visite. - Ne pas recevoir de visiteurs. - En cas de sortie, maintenir 2 mètres (6 pieds) entre vous et les autres.   Aussi:   - Se laver souvent les mains à l’eau et au savon pendant 20 secondes.   Les personnes qui sont malades ou qui reviennent de voyage doivent faire bien plus que pratiquer la distanciation.  Pour plus d’informations: <https://www.canada.ca/en/public-health/services/publications/diseases-conditions/self-monitoring-self-isolation-isolation-for-covid-19.html>  Nous apprécierions beaucoup que vous répondiez à quelques questions. Cliquez ici pour une courte enquête (5 questions) sur cette vidéo. |
